# Prognostic Enigma of Pancreatic Solid Pseudopapillary Neoplasm: A Single-Center Experience of 63 Patients

**DOI:** 10.3389/fsurg.2021.771587

**Published:** 2021-11-22

**Authors:** Xinbo Wang, Daojun Zhu, Wei Bao, Min Li, Sizhen Wang, Rongxi Shen

**Affiliations:** ^1^Research Institute of General Surgery, Jinling Hospital, Nanjing University Medical School, Nanjing, China; ^2^Department of Pathology, Jinling Hospital, Nanjing University Medical School, Nanjing, China

**Keywords:** malignant, pancreas, prognosis, solid pseudopapillary neoplasm, surgical management

## Abstract

**Background:** Studies investigating prognostic factors of solid pseudopapillary neoplasm (SPN) have been published with conflicting findings.

**Methods:** Retrospective analysis of 63 consecutive cases of SPN in our institution from January 2010 to December 2019 was carried out. The clinicopathological features, treatment practices along with survival associations were collected and analyzed.

**Results:** Fifteen patients (23.8%) were male, and 48 (76.2%) were female, with a median age of 34.0 ± 14.5 years. The larger tumor size was correlated with the more mixed components (*p* = 0.000) and the higher Ki-67 index (*p* = 0.042). No recurrence was found in the nine patients whose tumors fulfilled the WHO criteria for malignancy due to the presence of at least perineural invasion (6.4%), angiovascular invasion (2.3%), and/or adjacent organ invasion (6.4%). Microscopic infiltrative growth was detected in 9 (14.3%) tumors, which was correlated significantly with the WHO criteria (*p* = 0.002), capsule invasion (*p* = 0.005), and pancreatic parenchyma invasion (*p* = 0.001), but not with disease-free survival (*p* = 0.13). CD99 was found to be positively expressed in 88.9% (40/45) of tumors and more likely to have depressed Ki-67 index (*p* = 0.016). After a median follow-up of 58 months, only two patients (3.2%) had a recurrence after their first operation outside of our hospital. No patient died due to tumor progression.

**Conclusions:** Although survival is favorable with aggressive surgery, it is actually difficult to assess the prognostic factors of resected SPNs. Future investigations into the role of clinicopathological evaluation will unveil the prognostic enigma of pancreatic SPN after resection.

## Introduction

Solid pseudopapillary neoplasm (SPN) is a rare pancreatic tumor, which was first defined by Frantz in 1959 and classified as a potential malignant neoplasm by WHO in 2010 (1). The biologic behaviors of SPN are mostly less aggressive in spite of the tumor phenotype and classically present as large, solitary, well-circumscribed lesions with female predominance (2). However, up to 5–15% of patients demonstrate gross malignant features, such as invasion of adjacent organs or distant metastases, at the time of diagnosis or during the long-term follow-up after surgery (3). Because of its rarity and overall indolent but malignant course, SPN has not been well-studied, and standard staging tools have been questioned. Similarly, one of the biggest challenges in managing patients with SPN lies in predicting tumor behavior at the time of presentation (4). The WHO criteria of malignancy, which focused on microscopic features of malignancy, cannot be always equated with predicting the clinical malignant prognosis of SPN, nor were immunohistochemical stains, including the proliferative index Ki67 (5, 6).

Studies of investigating prognostic factors of SPN discuss plausible theories of the malignant potential of this enigmatic disease. Wu et al. (7) found that male patients had significantly poorer overall survival and disease-specific survival than female patients. Yin et al. (8) suggested that disrupted capsule, large tumor size, and pancreatic tail localization were viewed as the malignant SPN phenotype. Tang et al. (9) reported that peripancreatic lymphadenopathy on preoperative radiologic images was associated with malignancy in patients with SPN. Fu et al. (10) highlighted the proliferate index detected by Ki-67 in predicting the adverse outcome after an operation for SPN, but some study advocated the importance of pathologic evaluation in risk assessment in patients with SPNs (11, 12). Some authors disapproved the parenchyma-preserving resection on accounting of tumor recurrence (13), while others advocated function-preserving surgical approaches (14). Recent study based on the molecular alterations found that micro-RNA expression patterns in tumor might be able to predict metastatic spread of SPNs after surgery (15).

In this study, to validate the aforementioned prognostic factors usability in SPN and penetrate into the nature of this tumor, we described the clinicopathologic features, treatment practices, and outcome of 63 patients with SPNs in our institution.

## Materials and Methods

Data were extracted from the prospectively maintained databases of the patients operated for SPN at Research Institute of General Surgery, Jinling Hospital, Nanjing University Medical School, over a period of 10 years from January 2010 to December 2019. All 63 consecutive patients were identified and included in the study population. All patients underwent standard evaluation by routine blood investigations, including serum levels of carcinoembryonic antigen (CEA) and carbohydrate antigen 19-9 (CA19-9), and enhanced CT and/or MRI were examined before surgery. The type of resection was determined by the location and extent of the tumor with R0 resection as the primary aim. Minimally invasive and parenchyma sparing procedures would be performed in case that the definite diagnosis of SPN was reached through the preoperatively imaging and/or cytology. In particular, enucleations were carried out only when there was a safe distance between the tumor and the main pancreatic duct. According to the International Study Group on Pancreatic Surgery (ISGPS) classification (16), postoperative complications were defined as complications occurring within 30 days of surgery. Complications were graded as per the Clavien-Dindo classification (17). All of the tumors were pathologically confirmed to SPN by histological and immunohistochemical findings. Based on the WHO 2010 criteria, SPNs were classified as malignant if they showed invasion of either pancreatic tissue, peripancreatic nerves, or the vessels. Patients were followed up every 3 months for the first 1 year, every 6 months for the next year, and annually thereafter. Collected data included the clinicopathological and survival outcome from the hospital records and telephone interviews.

### Statistical Analysis

All statistical analyses were performed by SPSS ver. 21 (SPSS, Inc., IBM, Chicago, IL). Descriptive variables such as mean, SD, frequency, and rates were calculated. Statistical differences were detected using the independent samples *t*-test, Mann–Whitney *U*-test, or chi-square test when appropriate. Overall survival and disease-free survival were compared by Kaplan–Meier method.

## Results

### Patient Characteristics

It can be clearly found that the diagnosis of SPNs has progressively increased over 10 years in our institution, which is also in line with the reported significant increase in the incidence of SPN in the past decade. In total, 63 patients (15 males and 48 females) underwent surgical exploration for SPNs, ranging in age from 11 to 70 years (median 34 years). Demographic characteristics are described in Table 1. Nearly half of the patients (52.4%) had symptoms, of which abdominal pain was the most frequent (25.4%). Tumor markers, such as CA19-9, CA125, and CEA, were almost in the normal range. The localization of the tumor was in the head and neck in 25 patients (39.7%), including one with tumor recurrent *in situ*, and 38 in the body and tail (60.3%), including one with liver and retroperitoneal metastasis. The mean tumor size at diagnosis was 4.9 ± 2.4 cm in diameter. In the preoperative imaging (CT and/or MRI scan), most tumors (90.5%) were not accompanied by pancreatic duct dilatation. Tumors had radiologically heterogeneous (solid and cystic) features in 35 patients (55.5%). Solid features were found in 26 patients (41.3%), and cystic features were found in two patients (3.2%).

**Table 1:** Demographic characteristics of 63 patients with SPN.

**Clinical Features**	**Frequency (%) Mean ± SD**
**Gender**	
Male	15 (23.8)
Female	48 (76.2)
Age (years)	34.0 ± 14.5
BMI (Kg/m^2^)	22.5 ± 3.3
**Symptoms**	
No	30 (47.6)
Yes	33 (52.4)
Ca 19.9 >37 U/ml	2 (3.2)
**Tumor location**	
Head + neck	25 (39.7)
Body + tail	38 (60.3)
Size at radiology, cm	4.9 ± 2.4
**Radiologic features**	
Solid	26 (41.3)
Cystic	2 (3.2)
Mixed	35 (55.5)
Main pancreatic duct dilatation	6 (9.5)
Vascular encasement at radiology	1 (1.6)
Recurrence lesions at radiology	2 (3.2)
**Type of surgery**	
Enucleation	5 (7.9)
Distal pancreatectomy	22 (34.9)
Distal, spleen-preserving	14 (22.2)
Classical whipple	12 (19.1)
PPPD	3 (4.8)
Central pancreatectomy	5 (7.9)
Palliative surgery^*^	2 (3.2)
**Surgical complications**	
Hemorrhage	3 (4.8)
Pancreatic fistula	7 (11.1)
DGE	2 (3.2)
Pancreatitis	1 (1.6)
Postoperative hospital stay, days	14.6 ± 12.0

* *Palliative surgery includes metastasectomy and/or laparotomy*.

*PPPD, Pylorus-preserving pancreatoduodenectomy; DGE, Delayed gastric emptying*.

Sixty-one patients (96.8%) underwent curative surgery with negative margin (R0 resection). The type of operations included pancreaticoduodenectomy (15, 23.9%), distal pancreatectomy with splenectomy (22, 34.9%), and spleen-preserving distal pancreatectomy (15, 23.8%). Parenchyma-preserving procedures included central pancreatectomy (5, 7.9%) and tumor enucleation (5, 7.9%). No lymphadenectomy was performed. Notably, one patient was found to have lung squamous cell carcinoma before the surgery, so the simultaneous implementation of distal pancreatectomy and thoracoscopic lobectomy was performed. Only two patients (3.2%) who developed recurrence after their first operation outside of our hospital had debulking operations (R2). One patient who experienced retroperitoneal metastasis and peritoneal seeding after 5 years of distal pancreatectomy underwent metastasectomy. The other patient who experienced local recurrence in the pancreatic head after 7 years of tumor enucleation underwent laparotomy and tumor biopsy due to the encasement of the portal vein and its branches.

The median postoperative hospital stay was 14.6 days (ranging from 4 to 64 days). A total of 13 (20.6%) patients experienced postoperative complications. The most common morbidity following pancreatectomy was the postoperative pancreatic fistula (7, 11.1%). There were no reoperations or mortalities.

### Pathological and Immunohistochemistry Features

Pathological features are displayed in Table 2. Some selected clinicopathologic characteristics used to forecast recurrence were compared and are illustrated in Table 3. Of the 18 patients (28.6%) preoperatively misdiagnosed with other pancreatic neoplasms, the cystic neoplasms accounted for almost half of the preoperative misdiagnoses (8/18, 44.4%). SPN also had distinctive pathologic features, ranging from purely solid to purely cystic. It was noted that the larger tumor size was correlated significantly with the more mixed components (Table 3; *p* = 0.000) and the higher Ki-67 index (*p* < 0.042). Only one male patient was identified synchronous multifocal SPN by three tumors (3^*^3^*^2.8 cm, 1.3^*^1.2^*^0.8 cm, and 0.6^*^0.6^*^0.4 cm), which were all well-capsulated in the pancreatic head. Of note, 21 cases (33.3%) invaded the pancreatic capsule and 17 cases (27%) had pancreatic parenchyma invasion. According to the 2010 WHO classification for malignant SPNs, nine (14.3%) fulfilled inclusion criteria of the presence of at least one of the following: angiovascular infiltration (3.2%), perineural invasion (6.4%), and adjacent organ invasion (6.4%). None of these malignant characters was confirmed in the two recurrent patients at their pathological analysis of the primary tumor, but the metastasectomy specimens fulfilled WHO criteria for a malignant SPN: one with microscopic infiltrative growth pattern and perineural invasion, and the other one with adjacent organ invasion. Notably, microscopic infiltrative growth was detected in 9 (14.3%) patients and more likely to be identified as malignant (Table 3; *p* = 0.002). We also found that the microscopic infiltrative growth was correlated significantly with the capsule invasion (*p* = 0.005) and pancreatic parenchyma invasion (*p* = 0.001), but not with the perineural invasion or angiovascular invasion (*p* > 0.05). No lymph nodes were pathologically confirmed to be metastatic.

**Table 2:** Pathological features of the 63 resected SPN (at primary tumor operation).

**Clinical features**	**Frequency (%) Mean ± SD**
Size at gross pathology (cm)	5.3 ± 2.7
**Macroscopic features**	
Solid	26 (41.3)
Cystic	2 (3.2)
Mixed	35 (55.5)
Synchronous multifocal growth	1 (1.6)
**Microscopic growth pattern**	
Expansive growth	54 (85.7)
Infiltrative growth	9 (14.3)
Calcifications	6 (9.5)
Perineural invasion	4(6.4)
Angiovascular invasion	2(3.2)
Adjacent organ invasion	4(6.4)
Spleen	2(3.2)
Splenic vein/portal vein	1(1.6)
Duodenum	1(1.6)
Pancreatic parenchyma invasion	17 (27.0)
Capsule invasion	21 (33.3)

**Table 3:** Comparison of selected clinicopathologic characteristics used to forecast recurrence (at primary tumor operation).

**Characteristics**	**Tumor size**			**Microscopic growth pattern**			**CD99 stain**			**Total**
	**≤5 cm**	**>5 cm**	***P-value***	**Expansive**	**Infiltrative**	***P-value***	**Negative**	**Positive**	***P-value***	**(*n* = 63)**
	**(*n* = 36)**	**(*n* = 27)**		**(*n* = 54)**	**(*n* = 9)**		**(*n* = 5)**	**(*n* = 40)**		
Age (years)	36.7 ± 14.9	30.4 ± 13.5	0.473	34.4 ± 14.6	31.7 ± 14.8	0.616	37.8 ± 21.6	33.8 ± 14.2	0.459	34.0 ± 14.5
Tumor size (cm)	—	—	—	5.0 ± 2.6	4.3 ± 1.3	0.083	5.5 ± 2.4	5.0 ± 2.5	0.719	4.9 ± 2.4
Ki-67 (%)	2.7 ± 2.7	3.7 ± 3.8	**0.042**	3.3 ± 3.2	3.6 ± 4.6	0.276	5.0 ± 4.6	2.7 ± 2.7	**0.016**	3.3 ± 3.1
Gender			0.232			0.363			0.303	—
Male	11	4		12	3		0	12		15
Female	25	23		42	6		5	28		48
Tumor location			0.882			0.245			0.157	
Head + neck	14	11		20	5		2	16		25
Body + tail	22	16		34	4		3	24		38
WHO criteria			0.117			**0.002**			0.211	
Benign	33	21		50	4		3	34		54
Malignant	3	6		4	5		2	6		9
Macroscopic features			**0.000**			0.054			0.317	
Solid	22	4		20	6		1	17		26
Cystic	2	0		1	1		0	2		2
Mixed	12	23		33	2		4	21		35
Microscopic growth pattern			0.177						0.568	
Expansive growth	29	25		—	—	—	5	32		54
Infiltrative growth	7	2		—	—	—	0	8		9
Capsule invasion	10	11	0.28	14	7	**0.005**	2	13	0.737	21
Pancreatic parenchyma invasion	11	6	0.461	10	7	**0.001**	2	10	0.598	17
Perineural invasion	1	3	0.179	3	1	0.469	0	3	0.526	4
Angiovascular invasion	0	2	0.097	1	1	0.267	0	0	—	2

*Bold values indicate statistical significance*.

**Figure 1:**
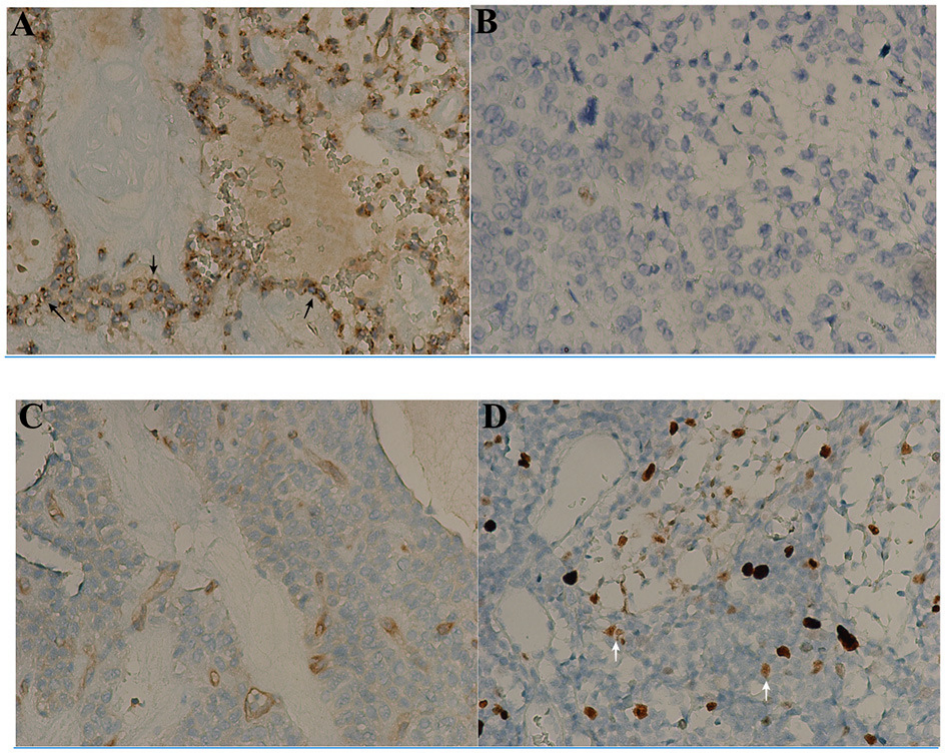
Immunohistochemical findings of CD99 and Ki-67 index in solid-pseudopapillary neoplasms (SPN) after surgery (x200). The tumor appears to have a particular dot-like paranuclei expression of CD99 (black arrow, **A**) with low Ki-67 index 1% **(B)**. The other tumor appearing to be negative for CD99 expression **(C)** has much high Ki-67 index 10% (white arrow, **D**).

Immunohistochemical analysis was performed in selected cases. The proliferation index (Ki-67) ranged from 0 to 15 (median, 3.3). Six cases showed Ki-67 index >5% and 3 cases >10%. β-Catenin and CD56 were positively expressed in 100% (47/47), CD10 in 89.5% (34/38), pancytokeratin in 52.3% (23/44), and neuron-specific enolase (NSE) in 66.7% (10/15). Chromogranin A was negative in 95.8% (44/46) of tumors, and synaptophysin negative in 37.3% (22/59) of tumors. In keeping with the known histological features of SPN, CD99 was found to be positively expressed in 88.9% (40/45) of tumors. Interestingly, the Ki-67 index of CD99-negative patients (Figure 1) was significantly higher than that of CD99-positive patients (Table 3; *p* = 0.016). In addition, the high female prevalence was reiterated with the positive expression of progesterone receptors in 93.1% (27/29) of tumors.

### Follow-up

**Figure 2:**
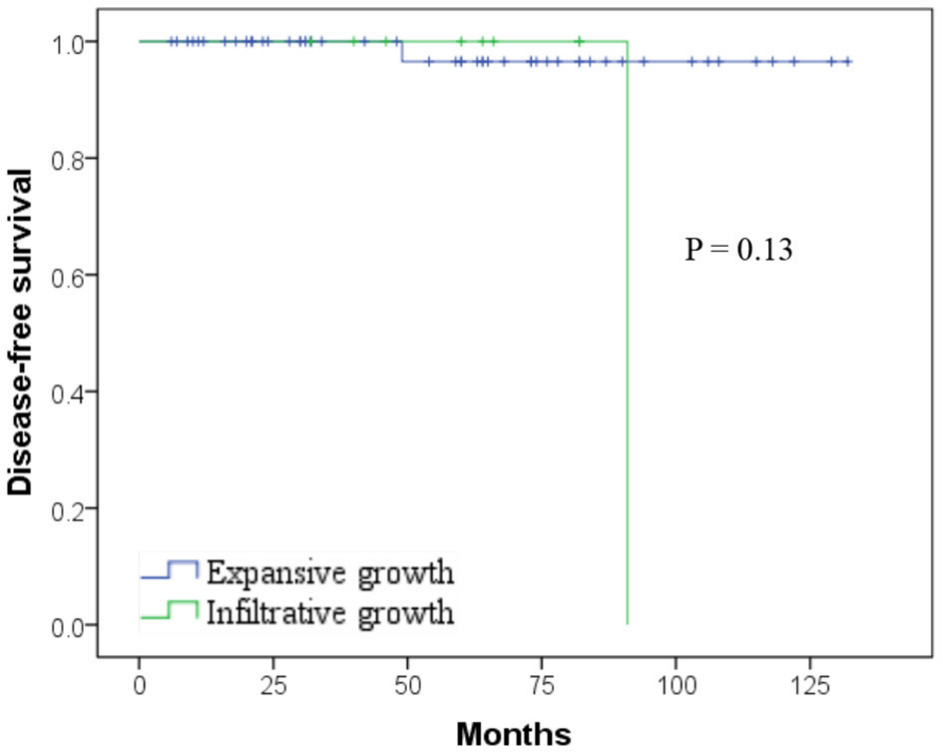
Kaplan–Meier curves showing no difference in disease-free survival (DFS) after surgery for solid pseudopapillary neoplasm (SPN) according to the microscopic infiltrative/expansive growth pattern (*p* = 0.13).

The final follow-up date was June 30, 2020. The median follow-up period was 58 months, ranging from 6 to 132 months. Except the female patient who died of lung cancer 16 months after simultaneous implementation of distal pancreatectomy and thoracoscopic lobectomy, no patient died of disease directly. Of the two recurred female patients, one was alive with locally advanced recurrent lesion in the pancreatic head at 96 months and the other one was alive with a single liver metastasis lesion after multiple subsequent metastasectomy at 124 months. There was no difference in disease-free survival (DFS) after surgery for SPN according to the microscopic infiltrative growth pattern or expansive growth pattern (Figure 2; *p* = 0.13), nor were all the clinical pathological and immunohistochemistry features, including the negative correlations between CD99 and Ki67 index. The 1-, 3-, and 5-year DFS rates were 100, 98.4, and 96.8%, respectively. The 1-, 3-, and 5-year overall survival (OS) rates were 100, 98.4, and 98.4%, respectively.

## Discussion

Because of the rarity of SPNs and the non-extensive case series present in literature, the biological behavior and risk factors for malignancy still failed to be clarified (3). The clinical data of the present series reinforce that the lack of histologic and clinical parameters to predict malignant behavior can also be pertaining to the favorable prognosis and long survival rate of SPNs.

Clinically, most studies reported that SPNs tended to occur in young female patients (18). However, our data were truly different as the ratio of male to female was 1:3.2, which was far fewer than the Western reports and similar to the Chinese reports (19). This difference possibly stems from fact that the incidence rate of male patients is rising and the difference of incidence rates comes from a different epidemiology of SPNs in different centers and regions. There was no difference in the age, tumor size, and location at diagnosis stroke between males and females. SPNs occurred more frequently in the body and tail of the pancreas. Nearly half of SPNs (52.4%) were incidentally discovered even though tumor size tended to be usually large with a median diameter of 5.3 cm, which is similar to other's reports (20). It was noted that the larger tumor size was correlated significantly with the more mixed components (*p* = 0.000) and the higher Ki-67 index (*p* < 0.042), not with the malignant potential (*p* = 0.117). The characteristic feature of SPN is the combination of solid pseudopapillae with fibrovascular stalks and cystic component with varied degeneration and hemorrhage (6). Some studies had shown a relationship between the tumor size >5 cm, tumor necrosis, high index of Ki-67, and SPNs with malignant potential (3, 6, 16). Our findings further corroborated that the combination of Ki-67 and the tumor size might be more informative than Ki-67 alone to predict the malignant potential (21).

Of note, one male patient in our cohort was identified synchronous multifocal SPN with three tumors well-capsulated macroscopically, which has not been reported in the literature so far. These multifocal tumors had perineural invasion with positive expression of β-catenin and CD56 and the high index of Ki-67 ranging from 10 to 15%. The pathogenesis of this tumor remains unclear. Kosmahl et al. (22) speculated that SPNs might originate from genital ridge-related cells, substantiated by the closeness of the genital ridges to the pancreatic anlage during embryogenesis. This might explain why one patient presented here has 18 years of menopause since 2002, whereas she suffered repeated recurrence and liver metastasis. The histogenesis of SPN remains unclear despite the extensive research using immunohistochemistry, electron microscopy, and molecular biology (23). Molecular and genetic studies on the synchronous multifocal SPN may reveal some new insights into the tumorigenesis of SPN.

The focus of this study was to evaluate the predictive value of the WHO criteria for malignant potential of SPNs from the perspective of surgical prognosis. The tumors of nine patients (14.3%) fulfilled the WHO criteria for malignancy, which was consistent with the data analyzed in large literature (24). Nevertheless, none of these nine patients experienced a recurrence. Despite a 100% negative predictive value, as the only two recurred patients did not accomplish those criteria in their primary tumors, the positive predictive value remains low. Some studies with large numbers of SPNs also failed to show a correlation between the microscopic malignant features and clinically evident malignance of SPNs (6, 24). In our study, microscopic infiltrative growth detected in nine (14.3%) patients was correlated significantly with the WHO criteria for malignance (*p* = 0.002), the capsule invasion (*p* = 0.005), and pancreatic parenchyma invasion (*p* = 0.001). Few literatures had previously studied the significance of infiltrative growth pattern in SPNs (12). Although infiltrative growth is not equal to malignant transformation from a low-grade to a high-grade malignancy or easy to relapse, it is related to the WHO malignant criteria and deserves attention. Some studies (25) have strived to delineate the pathologic features, including diffuse growth, extensive necrosis, dedifferentiation, and sarcomatoid features, necessary to its aggressive behavior or metastatic potential. It is essential to search for the high-grade morphologic features for identifying the aggressive SPNs. Our findings indicate that the accumulation of extensive clinical data is still required for this enigmatic disease.

The predictive value of Ki-67 index in evaluating the malignant potential of SPNs still remains controversial (21). The Ki-67 index higher than 5% may be a predictor of tumor recurrence in spite of its prognostic role not exploring or validating (26). Some studies regarded that Ki-67 was not associated with malignancy (3). The lack of signs of microscopic invasion in the surgical specimen should not discard a malignant potential of the tumor (27). In our cohort, the Ki-67 index was both <1% in the primary tumors in the only two recurred patients. One patient had the Ki-67 index 2% in the locally advanced recurrent lesions. And the other patient experiencing repeated recurrence and liver metastasis had Ki-67 index increased progressively with the increase in the number of metastases, from the initial tumor <1–15% of the last metastasectomy. Not only because Ki67% is a dynamic and evolutionary process as a proliferation marker protein, but also because it is highly expressed in proliferative cells, it is definitely not accurate to use Ki-67 alone as a predictor of tumor recurrence. Consistent with our finding, Walter et al. (28) also found that the Ki-67 index was 2% in the primary tumor, 10% in ovarian lesions, and 20% in liver metastases. It is recommended to review the assertive clinical features, such as local pancreatic or extrapancreatic invasion and metastases apart from the WHO microscopy criteria (1), since the clinicopathological features of SPN appear to be unreliable in assessing its biological behavior (5). Moreover, investigating the cause of the genetic variation of SPN may have enabled us to delineate the potential molecular pathways involved in recurrence (23).

The somewhat diverse immunohistochemical features of SPN lead to uncertainty in its origin (4). It is important to critically evaluate the diagnostic and prognostic utility of all the known markers. SPN appears to be highly unique expression of CD99 (29), and this dot-like paranuclei staining pattern was present in 88.9% (40/45), and the Ki-67 index of CD99-negative patients was significantly higher than that of CD99-positive patients (*p* = 0.016) in our series. There are few reports to investigate the role of CD99 expression in the prognostic value and efficacy assessment, so the evaluation value of immunohistochemistry in SPN may be deliberately emphasized. To our knowledge, the phenomenon of CD99 in downregulation of the proliferative activity by Ki-67 index has not been described in pancreatic SPNs to date. CD99 is involved in crucial biological processes, such as cell adhesion, migration, death, differentiation, immune responses, and tumorigenesis (30). Loss from the membrane and paranuclear localization of CD99 may result in the dysfunction of this adhesion molecule, and thus “discohesion” of SPN cells (31). However, CD99 plays an intriguing and dual role in different cell types. In particular, it corresponds to the cell malignancy or the oncosuppression in tumors (32). In gastric adenocarcinoma (33) and pulmonary carcinoid tumors (34), the decreased expression of CD99 was strongly associated with high proliferative activity, poor survival, and a heightened risk of metastasis formation, which may be related to increased migratory/invasion cell capabilities (35). Although the molecular basis underlying CD99 expression in SPN is still poorly understood, further investigations into the role of CD99 in the development of SPN are needed.

Another issue is the risk of parenchyma-preserving resection during the prognostic assessment for SPN recurrence. Few studies focus on analyzing an association of recurrence and initial type of operative procedure (2). One of the largest reports of 202 enucleations failed to provide information on the potential relationship between recurrence and the initial type of surgery (24). Although they did not analyze the recurrence events in relation to surgical procedures, the correlation between local approaches and non-radical resection seemed to be reasonable, according to the high risk for residual tumor or local recurrence (13). All the parenchyma-sparing surgery, including 5 (7.9%) in enucleation and 5 (7.9%) in central pancreatectomy, was performed radically (R0 resection) with no recurrence in our cohort. One of the two recurred patients experienced local recurrence 84 months after tumor enucleation in a county hospital and has survived for 96 months from the first surgery. This case further verified the conclusion that the local non-radical pancreatectomy for SPN was prone to recurrence (36).

The limitations to this study included the small number of cases and the retrospective nature, which might locate inconsistency in reporting pathologic data. In our cohort, none of the WHO criteria for malignance, infiltrative growth pattern, or the high Ki-67 index (≥4 or 5%) was confirmed in the primary tumor of the two recurred patients, but the metastasectomy specimens fulfilled some of the malignant characters. Moreover, the immunohistochemistry was heterogeneous due to using different panel of antibodies among different specimens, as CD99 was performed in selected cases (45/63). SPN was dismissed from mind by many pathologists and clinicians in the past, who focused much on diagnosis rather than clinicopathological characteristics with survival associations (14). Future research based on standardized clinicopathological evaluation of pancreatic SPN will be able to further unveil the prognostic enigma of pancreatic SPN after resection.

## Conclusion

Although aggressive surgery is the main method of management of pancreatic SPNs with favorable survival, it is generally difficult to assess the prognostic factors of resected SPNs. Our findings further corroborated that the current clinicopathological criteria for malignant potential SPNs should be deliberated. Future investigations into the role of clinicopathological evaluation will be able to further unveil the prognostic enigma of pancreatic SPN after resection.

## Data Availability Statement

The original contributions presented in the study are included in the article/supplementary material, further inquiries can be directed to the corresponding author/s.

## Ethics Statement

The studies involving human participants were reviewed and approved by the Ethics Committee of Jinling Hospital, Nanjing University Medical School. The patients/participants provided their written informed consent to participate in this study.

## Author Contributions

XW was involved in the identification, selection, and management of patient cases, and wrote and revised the manuscript. DZ, ML, SW, and RS was involved in the management of patient cases and data acquisition, analysis, and interpretation. WB performed the histological images analysis and reviewed the manuscript. All authors read and approved the final manuscript.

## Conflict of Interest

The authors declare that the research was conducted in the absence of any commercial or financial relationships that could be construed as a potential conflict of interest.

## Publisher's Note

All claims expressed in this article are solely those of the authors and do not necessarily represent those of their affiliated organizations, or those of the publisher, the editors and the reviewers. Any product that may be evaluated in this article, or claim that may be made by its manufacturer, is not guaranteed or endorsed by the publisher.

## References

[1] Bosman FT Carneiro F Hruban RH. WHO Classification of Tumours of the Digestive System. Lyon: 4th edn. Vol. 3. World Health Organization (2010).31433515

[2] Hanada K Kurihara K Itoi T Katanuma A Sasaki T Hara K. Clinical and pathological features of solid pseudopapillary neoplasms of the pancreas: a nationwide multicenter study in Japan. Pancreas. (2018) 47:1019–26. 10.1097/MPA.0000000000001114 30059473

[3] Kang CM Choi SH Kim SC Lee WJ Choi DW Kim SW. Predicting recurrence of pancreatic solid pseudopapillary tumors after surgical resection: a multicenter analysis in Korea. Ann Surg. (2014) 260:348–55. 10.1097/SLA.0000000000000583 24743622

[4] La Rosa S Bongiovanni M. Pancreatic solid pseudopapillary neoplasm: key pathologic and genetic features. Arch Pathol Lab Med. (2020) 144:829–37. 10.5858/arpa.2019-0473-RA 31958381

[5] Naar L Spanomichou D Mastoraki A Smyrniotis V Arkadopoulos N. Solid pseudopapillary neoplasms of the pancreas: a surgical and genetic enigma. World J Surg. (2017) 41:1871–81. 10.1007/s00268-017-3921-y 28251269

[6] Jutric Z Rozenfeld Y Grendar J Hammill CW Cassera MA Newell PH. Analysis of 340 patients with solid pseudopapillary tumors of the pancreas: a closer look at patients with metastatic disease. Ann Surg Oncol. (2017) 24:2015–22. 10.1245/s10434-017-5772-z 28299507

[7] Wu J Mao Y Jiang Y Song Y Yu P Sun S. Sex differences in solid pseudopapillary neoplasm of the pancreas: a population-based study. Cancer Med. (2020) 9:6030–41. 10.1002/cam4.3180 32578384 PMC7433837

[8] Yin Q Wang M Wang C Wu Z Yuan F Chen K. Differentiation between benign and malignant solid pseudopapillary tumor of the pancreas by MDCT. Eur J Radiol. (2012) 81:3010–8. 10.1016/j.ejrad.2012.03.013 22520082

[9] Tang X Zhang J Che X Chen Y Wang C. Peripancreatic lymphadenopathy on preoperative radiologic images predicts malignancy in pancreatic solid pseudopapillary neoplasm. Int J Clin Exp Med. (2015) 8:16315–21. 26629150 PMC4659038

[10] Yang F Yu X Bao Y Du Z Jin C Fu. Prognostic value of Ki-67 in solid pseudopapillary tumor of the pancreas: Huashan experience and systematic review of the literature. Surgery. (2016) 159:1023–31. 10.1016/j.surg.2015.10.018 26619927

[11] Estrella J Li L Rashid A Wang H Katz M Fleming J. Solid pseudopapillary neoplasm of the pancreas: clinicopathologic and survival analyses of 64 cases from a single institution. Am J Clin Path. (2014) 38:147–57. 10.1097/PAS.0000000000000141 24418850

[12] Marchegiani G Andrianello S Massignani M Malleo G Maggino L Paiella S. Solid pseudopapillary tumors of the pancreas: specific pathological features predict the likelihood of postoperative recurrence. J Surg Oncol. (2016) 114:597–601. 10.1002/jso.24380 27471041

[13] Tjaden C Hassenpflug M Hinz U Klaiber U Klauss M Buchler MW. Outcome and prognosis after pancreatectomy in patients with solid pseudopapillary neoplasms. Pancreatology. (2019) 19:699–709. 10.1016/j.pan.2019.06.008 31227367

[14] Liu M Liu J Hu Q Xu W Liu W Zhang Z. Management of solid pseudopapillary neoplasms of pancreas: a single center experience of 243 consecutive patients. Pancreatology. (2019) 19:681–5. 10.1016/j.pan.2019.07.001 31281058

[15] Cohen SJ Papoulas M Graubardt N Ovdat E Loewenstein S Kania-Almog J. Micro-RNA expression patterns predict metastatic spread in solid pseudopapillary neoplasms of the pancreas. Front Oncol. (2020) 10:328. 10.3389/fonc.2020.00328 32232006 PMC7082878

[16] Bassi C Marchegiani G Dervenis C Sarr M Abu Hilal M Adham M. The 2016 update of the International Study Group (ISGPS) definition and grading of postoperative pancreatic fistula: 11 years after. Surgery. (2017) 161:584–91. 10.1016/j.surg.2016.11.014 28040257

[17] Dindo D Demartines N Clavien PA. Classification of surgical complications: a new proposal with evaluation in a cohort of 6336 patients and results of a survey. Ann Surg. (2004) 240:205–13. 10.1097/01.sla.0000133083.54934.ae 15273542 PMC1360123

[18] Huffman BM Westin G Alsidawi S Alberts SR Nagorney DM Halfdanarson TR. Survival and prognostic factors in patients with solid pseudopapillary neoplasms of the pancreas. Pancreas. (2018) 47:1003–7. 10.1097/MPA.0000000000001112 30036214

[19] Wang P Wei J Wu J Xu W Chen Q Gao W. Diagnosis and treatment of solid-pseudopapillary tumors of the pancreas: a single institution experience with 97 cases. Pancreatology. (2018) 18:415–9. 10.1016/j.pan.2017.12.012 29548800

[20] Xu Y Zhao G Pu N Nuerxiati A Ji Y Zhang L. One hundred twenty-one resected solid pseudopapillary tumors of the pancreas: an 8-year single-institution experience at Zhongshan hospital, shanghai, China. Pancreas. (2017) 46:1023–8. 10.1097/MPA.0000000000000885 28742543

[21] Yang F Wu W Wang X Zhang Q Bao Y Zhou Z. Grading solid pseudopapillary tumors of the pancreas: the Fudan prognostic index. Ann Surg Oncol. (2021) 28:550–9. 10.1245/s10434-020-08626-z 32424583

[22] Kosmahl M Seada L Jänig U Harms D Klöppel G. Solid–pseudopapillary tumor of the pancreas: its origin revisited. Virchows Arch. (2000) 436:473–80. 10.1007/s004280050475 10881741

[23] Guo M Luo G Jin K Long J Cheng H Lu Y. Somatic genetic variation in solid pseudopapillary tumor of the pancreas by whole exome sequencing. Int J Mol Sci. (2017) 18:81. 10.3390/ijms18010081 28054945 PMC5297715

[24] Law JK Ahmed A Singh VK Akshintala VS Olson MT Raman SP. A systematic review of solid-pseudopapillary neoplasms: are these rare lesions? Pancreas. (2014) 43:331–7. 10.1097/MPA.0000000000000061 24622060 PMC4888067

[25] Watanabe Y Okamoto K Okada K Aikawa M Koyama I Yamaguchi H. A case of aggressive solid pseudopapillary neoplasm: comparison of clinical and pathologic features with non-aggressive cases. Pathol Int. (2017) 67:202–7. 10.1111/pin.12516 28208222

[26] Yu P Cheng X Du Y Yang L Xu Z Yin W. Solid pseudopapillary neoplasms of the pancreas: a 19-year multicenter experience in China. J Gastrointest Surg. (2015) 19:1433–40. 10.1007/s11605-015-2862-8 26001371

[27] Geers C Moulin P Gigot JF Weynand B Deprez P Rahier J. Solid and pseudopapillary tumor of the pancreas-review and new insights into pathogenesis. Am J Surg Pathol. (2006) 30:1243–9. 10.1097/01.pas.0000213311.28682.b2 17001154

[28] Walter T Hommell-Fontaine J Hervieu V Adham M Poncet G Dumortier J. Primary malignant solid pseudopapillary tumors of the gastroduodenal area. Clin Res Hepatol Gastroenterol. (2011) 35:227–33. 10.1016/j.clinre.2011.01.004 21345760

[29] Guo Y Yuan F Deng H Wang HF Jin XL Xiao JC. Paranuclear dot-like immunostaining for CD99: a unique staining pattern for diagnosing solid-pseudopapillary neoplasm of the pancreas. Am J Surg Pathol. (2011) 35:799–806. 10.1097/PAS.0b013e318219c036 21566515

[30] Pasello M Manara MC Scotlandi K. CD99 at the crossroads of physiology and pathology. J Cell Commun Signal. (2018) 12:55–68. 10.1007/s12079-017-0445-z 29305692 PMC5842202

[31] Li L Li J Hao C Zhang C Mu K Wang Y. Immunohistochemical evaluation of solid pseudopapillary tumors of the pancreas: the expression pattern of CD99 is highly unique. Cancer Lett. (2011) 310:9–14. 10.1016/j.canlet.2011.04.017 21775056

[32] Manara MC Pasello M Scotlandi K. CD99: a cell surface protein with an oncojanus role in tumors. Genes. (2018) 9:159. 10.3390/genes9030159 29534016 PMC5867880

[33] Lee JH Kim SH Wang LH Choi YL Kim YC Kim JH. Clinical significance of CD99 down-regulation in gastric adenocarcinoma. Clin Cancer Res. (2007) 13:2584–91. 10.1158/1078-0432.CCR-06-1785 17473187

[34] Pelosi G Fraggetta F Sonzogni A Fazio N Cavallon A Viale G. CD99 immunoreactivity in gastrointestinal and pulmonary neuroendocrine tumours. Virchows Arch. (2000) 437:270–4. 10.1007/s004280000240 11037347

[35] Zucchini C Manara MC Pinca RS De Sanctis P Guerzoni C Sciandra M. CD99 suppresses osteosarcoma cell migration through inhibition of ROCK2 activity. Oncogene. (2014) 33:1912–21. 10.1038/onc.2013.152 23644663

[36] The European Study Group on Cystic Tumours of the Pancreas. European evidence-based guidelines on pancreatic cystic neoplasms. Gut. (2018) 67:789–804. 10.1136/gutjnl-2018-316027 29574408 PMC5890653

